# Decreased expression of CDH1 or CTNNB1 affects poor prognosis of patients with esophageal cancer

**DOI:** 10.1186/s12957-016-0956-8

**Published:** 2016-09-06

**Authors:** Hideyuki Ishiguro, Takehiro Wakasugi, Yukio Terashita, Nobuhiro Sakamoto, Tatsuya Tanaka, Koji Mizoguchi, Hiroyuki Sagawa, Tomotaka Okubo, Hiromitsu Takeyama

**Affiliations:** Gastroenterological Surgery, Nagoya City University Graduate School of Medical Science, 1 Kawasumi, Mizuho-cho, Mizuho-ku, Nagoya, 467-8601 Japan

**Keywords:** CDH1-CTNNB1, Prognosis, Immunohistochemistry, Esophageal cancer

## Abstract

**Background:**

E-cadherin/CDH1 is one of the proteins involved in cell adhesion, and it is known that decreased expression of E-cadherin induces lymph node metastasis in esophageal cancer. Beta catenin/CTNNB1, which is an important component of the Wnt signaling pathway, binds to E-cadherin at the cell membrane, where the complex of these two proteins functions in the stabilization of cell adhesion. However, its role in the pathogenesis of esophageal cancer is still unknown.

**Methods:**

This study included 86 patients with esophageal cancer who underwent surgery between 1998 and 2007. The expression of the *E-cadherin/CDH1* gene product (E-cadherin/CDH1) and that of the beta catenin/CTNNB1 protein in the cell membrane were analyzed by immunohistochemistry. We examined the correlations among CDH1 or CTNNB1 expression, clinicopathological factors, and the prognosis of patients with ESCC.

**Results:**

CDH1 and CTNNB1 were expressed in 52.3 % (45/86) and 36.0 % (31/86) of tumor samples, respectively. Both CDH1 and CTNNB1 were co-expressed in 22.1 % (19/86) of esophageal cancer tissues. CDH1 expression correlated with the *p*-stage (stages I–II vs stages III–IV, *p* = 0.032), *T* factor (T1–2 vs T3–4, *p* = 0.0088), and lymphatic invasion (*p* = 0.019). However, CDH1 expression did not correlate with the *N* factor or the blood vessel invasion. CTNNB1 expression correlated with the *T* factor (T1–2 vs T3–4, *p* = 0.0015), *p*-stage (stages I–II vs stages III–IV, *p* = 0.030), and lymphatic invasion (*p* = 0.007). The CDH1(+)/CTNNB1(+) phenotype was inversely correlated with the *T* factor, *N* factor, *p*-stage, lymphatic invasion, and blood vessel invasion. Furthermore, patients whose tumors were double-positive for CDH1 and CTNNB1 had a significantly higher survival rate than those whose tumors were negative for CDH1 or CTNNB1 (log-rank test, *p* = 0.0192). The *T* factor and *N* factor had a strong negative correlation with double-positive tumors. These were both independent prognostic factors, as was the double-positive phenotype. A univariate analysis indicated that the *T* factor, the *N* factor, and CDH1 and CTNNB1 co-expression were significant variables that predicted survival (hazard ratio, 2.387; 95 % confidence interval, 1.115–5.102; *p* = 0.025).

**Conclusions:**

Decreased expression of CDH1 or CTNNB1 in the cell membranes of cancer cells is associated with poor survival of patients with esophageal cancer.

## Background

The prognosis of patients with esophageal cancer remains poor, which highlights the need for the development of new treatment strategies. Today, the overall 5-year survival rate is less than 50 % despite the use of multimodal therapies. To develop novel treatment strategies, it is important to understand the biological behavior of esophageal cancer. Recent studies have found that several genes and molecules are involved in the origin and/or progression of esophageal cancer, including TP53 [1], *deleted in esophageal cancer 1* (*DEC1*) [2], *deleted in colorectal cancer* (*DCC*) [3], *deleted in lung cancer 1* (*DLC1*) [4], cyclinD1 [5], and adenomatous polyposis coli (APC) [6]. However, the precise mechanisms that underlie the development and progression of esophageal squamous cell carcinoma (ESCC) are still unclear.

The *E-cadherin/CDH1* gene product is a transmembrane protein that is involved in cell adhesion in normal epithelia [7–9]. E-cadherin is also involved in the stabilization of cell adhesion in normal cells and in signal transduction through connections with beta catenin [10]. Beta catenin/CTNNB1, which is a major component of the Wnt signaling pathway, plays an important role in the carcinogenesis of various malignancies. Beta catenin also functions in cell-to-cell adhesion [11–14].

Decreased expression of CDH1 is associated with the poor prognosis of patients with esophageal cancer [15, 16]. However, the involvement of decreased expression of CTNNB1 at the cell membrane is unclear in metastasis, and the progression of esophageal cancer is unclear.

In this paper, we investigated the clinicopathological significance of *CDH1 and CTNNB1* protein expression in the cell membrane and the relation between the co-expression of CDH1 and CTNNB1 in 86 patients with resectable ESCC.

## Methods

### Tissue samples

Samples were obtained from 86 patients with ESCC who underwent surgery at the Department of Gastroenterological Surgery, Nagoya City University Medical School between 1997 and 2005. None of the patients received pre-operative chemotherapy or radiation. The tumors were classified according to the sixth UICC guidelines for clinical and pathological studies on carcinoma of the esophagus. R0/R1 resection was performed on all patients. T4 cases included combined resection of 6 pleuras, 3 pericardia, 11 thoracic ducts, and 4 tracheas because of the invasion of the structure. Stage IV cases included 20 M1a (cervical lymph node metastasis of Ut cases and celiac lymph node metastasis of Lt) and 14M1b (10 non-regional lymph node metastases of Mt cases and 4 lung metastases). We performed combined resection of the partial lung in lung metastasis cases.

### Immunohistochemistry

Immunohistochemical staining was performed on formalin-fixed, paraffin-embedded primary human ESCC tissues using monoclonal anti-E-cadherin (Dako, CA, USA) or anti-beta catenin antibodies (BD Biosciences, Lexington, KY, USA) at dilutions of 1:50 and 1:500, respectively. Paraffin-embedded sections of tumors were deparaffinized, rehydrated, heat-treated by microwaving in 10 mM citrate buffer for 15 min for antigen retrieval, and cooled to room temperature. The sections were then treated with 0.3 % H_2_O_2_ in methanol for 30 min to neutralize endogenous peroxidase activity, after which the sections were blocked with normal goat serum for 10 min. Next, the slides were incubated with antibody H-100 overnight at room temperature in a humidified chamber. Immunoreactive proteins were detected by a DAKO Envision System using HRP and DAB, which was followed by a hematoxylin counterstain. Immunostaining for E-cadherin/CDH1 and beta catenin/CTNNB1 was subjectively assessed by two independent investigators (HI and TT), and discordant results were resolved by consultation with a third investigator (TW). In regard to the evaluation of CDH1 expression, immunostaining was considered positive only when unequivocally strong staining of the cell membrane was present in more than 50 % of the tumor cells, as analyzed by light microscopy. Cases with only faint staining were regarded as negative. For the evaluation of CTNNB1 expression, immunostaining was scored as positive only when unequivocally strong staining of the cell membrane was present in more than 50 % of the tumor cells similar to CDH1. Cases with only faint staining were regarded as negative.

### Statistical analysis

The chi-squared test was used to compare the correlations between clinicopathological factors and the expression of CDH1 and CTNNB1. The cumulative survival rates were calculated according to the Kaplan-Meier method and were compared by the Cox-Mantel test. A multivariate analysis by the Cox proportional hazard risk model was used to obtain the conditional risk of death due to ESCC. Differences were considered statistically significant when *p* values were less than 0.05.

## Results

### The correlation of clinicopathological factors with the expression of CDH1 and CTNNB1

**Fig. 1:**
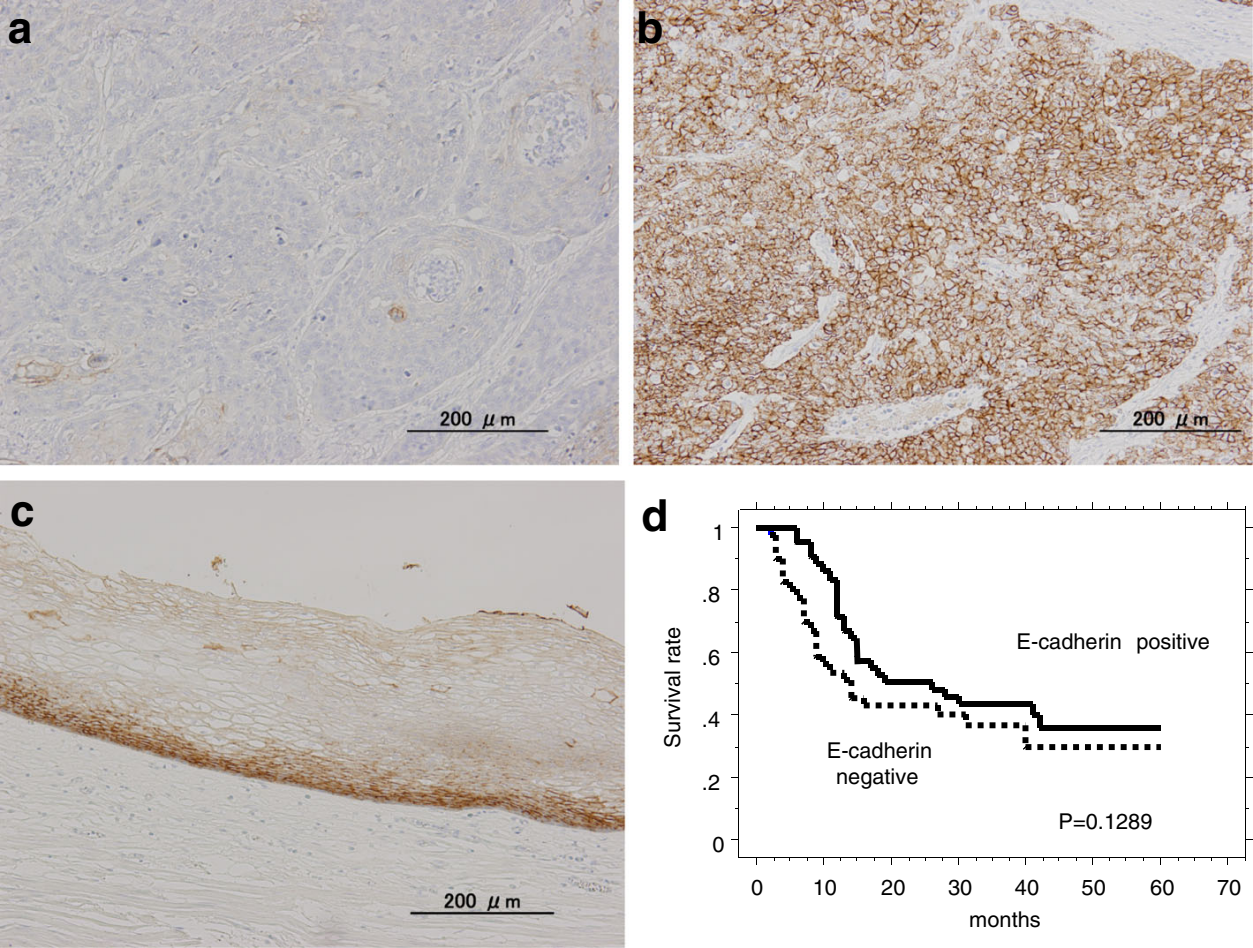
Representative immunostains for CDH1 (×100). **a** Negative CDH1 staining in the cell membrane of tumor cells. **b** Positive CDH1 staining in the cell membrane of tumor cells. **c** Expression of CDH1 in normal esophageal mucosa. **d** Kaplan-Meier survival curve for patients with esophageal cancer whose tumors were classified as either positive or negative for CDH1 expression by IHC. CDH1 status did not demonstrate a significant (log-rank, *p* = 0.1289) relation with patient survival

**Fig. 2:**
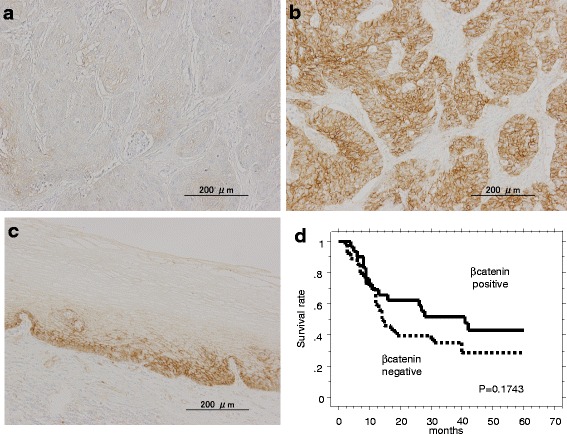
Representative immunostains for CTNNB1 (×100). **a** Negative CTNNB1 staining in the cell membrane of tumor cells. **b** Positive CTNNB1 staining in the cell membrane of tumor cells. **c** Expression of CTNNB1 in normal esophageal mucosa. **d** Kaplan-Meier survival curve for patients with esophageal cancer whose tumors were classified as either positive or negative for CTNNB1 by IHC. CTNNB1 status did not demonstrate a significant (log-rank, *p* = 0.1743) relation with patient survival

First, we investigated the expression of the CDH1 and CTNNB1 proteins in ESCC tissues by immunohistochemistry. Representative images of cancer tissues immunostained for CDH1 and CTNNB1 are shown in Fig. 1a, b and in Fig. 2a, b, respectively. In normal tissue, both CDH1 and CTNNB1 are strongly expressed. Typical positive cells showed diffuse staining of CDH1 and CTNNB1 in the cell membrane. Immunostaining for CDH1 and CTNNB1 was positive in 52.3 % (45/86) and 36.0 % (31/86) of patients, respectively (Table 1). CDH1 expression correlated with the *p*-stage (stages I–II vs stages III–IV, *p* = 0.032), *T* factor (T1–2 vs T3–4, *p* = 0.0088), and lymphatic invasion (*p* = 0.019). However, it did not correlate with the *N* factor or the *v* factor. CTNNB1 expression correlated with the *p*-stage (stages I–II vs stages III–IV, *p* = 0.030) and Ly factor (*p* = 0.007) (Table 1). Both CDH1 and CTNNB1 were co-expressed in 22.1 % (19/86) of esophageal cancer tissues. The CDH1(+)/CTNNB1(+) phenotype was inversely correlated with the *T* factor, *N* factor, *p*-stage, lymphatic invasion, and blood vessel invasion (Table 2). Additionally, no correlations were observed between the expression of CDH1 and that of CTNNB1 (data not shown).

**Table 1:** Correlation of CDH1 and CTNNB1 expression by IHC with clinicopathological factors, including patient and tumor characteristics, in esophageal cancer

		No. of patients (*n* = 86)			
Characteristics	Case	CDH1(+)	*p* value	CTNNB1(+)	*p* value
Age at surgery					
<65 years	50	30		18	
>65 years	36	15	0.009	13	0.991
Gender					
Male	69	40		26	
Female	17	5	0.035	5	0.525
Tumor status					
T1	17	13		10	
T2	8	5		5	
T3	37	17		9	
T4	24	10		7	
T1–2 vs T3–4			0.0088		0.0015
Lymph node status					
N0	18	11		10	
N1	68	34		21	
N0 vs N1			0.40		0.053
Pathological stage					
I	13	9		8	
II	11	8		5	
III	28	15		8	
IV	34	13		10	
I–II vs III–IV			0.032		0.029
Lymphatic invasion					
Negative	16	13		10	
Positive	54	26		14	
Unknown	16		0.019		0.007
Blood vessel invasion					
Negative	30	19		13	
Positive	40	20		11	
Unknown	16		0.266		0.167

**Table 2:** Correlation of CDH1 and CTNNB1 expression by IHC with clinicopathological factors, including patient and tumor characteristics, in esophageal cancer

		No. of patients (*n* = 86)	
Characteristics	Case	CDH1(+)/CTNNB1(+)	*p* value
Age at surgery			
<65 years	50	14	
>65 years	36	5	0.119
Gender			
Male	69	17	
Female	17	2	0.252
Tumor status			
T1	17	8	
T2	8	5	
T3	37	2	
T4	24	4	
T1 vs T2–4			0.006
Lymph node status			
N0	18	8	
N1	68	11	
N0 vs N1			0.010
Pathological stage			
I	13	6	
II	11	5	
III	28	3	
IV	34	5	
I–II vs III–IV			0.001
Lymphatic invasion			
Negative	16	9	
Positive	54	7	
Unknown	16		0.003
Blood vessel invasion			
Negative	30	11	
Positive	40	5	
Unknown	16		0.020

### Survival curves and the expression of CDH1 and CTNNB1

**Fig. 3:**
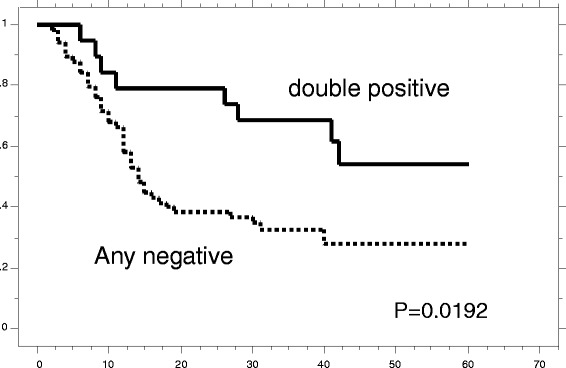
**a** Kaplan-Meier survival curve for patients with esophageal cancer whose tumors were classified as either positive or negative for CDH1 and CTNNB1 expression by IHC. Double-positive staining status was found to be strongly associated (log-rank, *p* = 0.0192) with patient survival

Next, we investigated the correlation between positive staining for CDH1 and CTNNB1 and the survival of patients with ESCC after surgery. Neither CDH1 nor CTNNB1 exerted a significant effect on patient survival (Figs. 1d and 2d). Indeed, patients whose tumors were positive for CDH1 by IHC did not demonstrate a significantly longer survival after surgery than patients whose tumors were negative (26.7 ± 2.29 months (*n* = 45) vs 20.9 ± 2.59 months (*n* = 41), respectively; *p* = 0.1289 by Log-rank test; Fig. 1d). Moreover, no significant differences were observed with respect to survival after surgery between patients whose tumors were positive and patients whose tumors were negative for CTNNB1 (27.9 ± 2.97 months (*n* = 31) vs 21.5 ± 2.07 months (*n* = 55), respectively; *p* = 0.1743 by Log-rank test; Fig. 2d). However, the co-expression of CDH1 and CTNNB1 was associated with a significantly longer survival after surgery compared with patients with negative tumors (*p* = 0.0192) (Fig. 3).

A univariate analysis showed that, among the clinicopathological factors examined in this study, the extent of the primary tumor (risk ratio, 6.289; *p* < 0.001), lymph node metastasis (risk ratio, 5.812; *p* < 0.001), lymphatic invasion (risk ratio, 5.917; *p* = 0.003), blood vessel invasion (risk ratio, 3.135; *p* = 0.002), and positive immunostaining for CDH and CTNNB1 (risk ratio, 2.387; *p* = 0.025) were statistically significant prognostic factors (Table 3). A multivariate analysis revealed that CDH1 and CTNNB1 expression was not an independent prognostic factor (data not shown).

**Table 3:** Univariate analysis

Parameter	Risk ratio	95 % CI	*p* value
Age at surgery			
<65 years	1		
>65 years	1.573	0.907–2.729	0.107
Gender			
Female	1		
Male	1.125	0.563–2.247	0.739
Primary tumor			
T1–3	1		
T4	6.289	3.425–11.49	<0.001
Lymph node metastasis			
N0	1		
N1	5.812	2.088–16.39	<0.001
Lymphatic invasion			
Negative	1		
Positive	5.917	1.812–19.23	0.003
Venous invasion			
Negative	1		
Positive	3.135	1.541–6.369	0.002
Double-positive immunostaining			
Negative	1		
Positive	2.387	1.115–5.102	0.025

*CI* confidence interval

## Discussion

The loss of cellular adhesion results in increased invasiveness and metastatic ability of various types of cancer cells. Reduced cellular adhesion has been linked to susceptibility to cancer in some cell types. CDH1 and CTNNB1 are two of the most important determinants of cell polarity and intercellular adhesion. Additionally, the CDH1 and CTNNB1 complex is involved in the maintenance of cell-cell adhesion [10]. In normal cells, the complex is localized to the cell membrane [17, 18]. Consistent with this finding, our current experiments showed that both CDH1 and CTNNB1 were expressed in the cell membrane in noncancerous cells (Figs. 1c and 2c). Reduced expression of the cadherin-catenin complex in various carcinomas has been reported, and the decreased expression of these proteins has been found to be correlated with high grade and advanced tumor stage, including esophageal carcinoma [10, 19].

However, it is still unclear how CDH1 expression is regulated in ESCC. Some factors such as hypermethylation [20], microRNA [21, 22], and PDX1 expression [23], which have been shown to regulate the expression of CDH1, should be investigated in future studies.

The cytoplasmic and nuclear expression of CTNNB1 is controlled by Wnt-1, APC, and axis inhibitor 1/AXIN1 [24, 25]. Most of the mutations in *APC* result in a truncated APC protein, which can form a complex with but cannot degrade CTNNB1 [24, 26]. The CTNNB1 level in the nucleus may also be increased by mutations in the CTNNB1 gene itself, which may interfere with normal protein degradation [27, 28]. Thus, a number of mechanisms result in an increased level of CTNNB1, including mutations in the CTNNB1 gene and truncated APC [27, 28]. However, to the best of our knowledge, the mechanism by which CTNNB1 expression is decreased in the cell membrane is still unclear. Few mutations in CTNNB1 and APC occur in the setting of esophageal cancer [29, 30]. We previously reported that the accumulation of CTNNB1 in the nucleus occurs infrequently in esophageal cancer [31].

The CDH1 gene and the CTNNB1 gene are localized to human chromosomes 16q22.11 [32] and 3p21 [33], respectively. Many studies have suggested that the *CDH1* locus (16q) and the CTNNB1 locus (3p) may harbor tumor suppressor genes for prostate cancer [34] and bladder cancer [35]. Therefore, the loss of CDH1 may also contribute to the development of many other types of cancers. Further studies are required to determine whether chromosomal losses occurred in the CDH1 and CTNNB1 loci in the esophageal tumor tissues that were examined in this study.

We analyzed the expression of CDH1 and CTNNB1 by immunohistochemistry. While our results suggested that CDH1 or CTNNB1 expression alone did not affect the prognosis of patients, whether CDH1 and CTNNB1 expression may serve as a good prognostic marker in esophageal cancer is still controversial. In the meta-analysis, there are two reports that CDH1 expression alone is a valid prognostic marker [36] and aberrant CTNNB1 alone is a prognostic factor [37]. Our data suggested that CDH alone or CTNNB1 alone did not affect the prognosis of the patients with esophageal cancer, though we could not clarify the discrepancy between their data and ours.

Some clinical studies have reported that CDH1 expression is an indicator of poor prognosis or malignant potential in gastric cancer [38], breast cancer [39], and non-small cell lung cancer [40].

In this study, we found that the decreased expression of CDH1 or CTNNB1 in the cell membrane in cancer tissues accompanied the local progression and lymph node metastasis of esophageal cancer (Table 2). In addition, patients with lower CDH1 or CTNNB1 expression had a poorer prognosis (Fig. 3). Our data suggested co-expression of CDH1 and CTNNB1 in the cell membrane might be needed for cell stability because cell instability often causes malignant change of the cancer cell.

In patients with esophageal cancer, many prognostic markers, including cyclinD1 and mouse double minute 2 homolog (MDM2), have been reported [41, 42]. Furthermore, we have also reported that *survivin* [43], *pituitary tumor transforming gene 1* (*PTTG1*) [44], *DNA fragmentation factor 45* (*DFF45*) [45], and *DROSHA* [46] may be prognostic markers of ESCC*.* Thus, the decreased expression of CDH1 or CTNNB1 represents an additional potential prognostic indicator for patients with ESCC.

Although the precise molecular mechanisms through which CDH1 or CTNNB1 is downregulated still need to be clarified, our data clearly indicated that the downregulation of CDH1 or CTNNB1 may be a prognostic marker for ESCC. Finally, these proteins may serve as molecular targets for the development of effective therapeutic agents for patients with esophageal cancer.

## Conclusions

Decreased expression of CDH1 or CTNNB1 in the cell membranes of cancer cells is associated with poor survival of patients with esophageal cancer.

## Abbreviations

APC, adenomatous polyposis coli; AXIN1, axis inhibitor 1; CDH1, cadherin 1; CTNNB1, catenin, beta-1; DCC, deleted in colorectal cancer; DEC1, deleted in esophageal cancer 1; DFF45, DNA fragmentation factor 45; DLC1, deleted in lung cancer 1; ESCC, esophageal squamous cell carcinoma; MDM2, mouse double minute 2 homolog; PTTG1, pituitary tumor transforming gene 1
